# Ileitis

**Published:** 1869-10

**Authors:** S. P. Breed

**Affiliations:** Princeton, Ill.


CHICAGO MEDICAL EXAMINER.

N. S. DAVIS, M.D., Editor.

VOL. X. OCTOBER, 1869. NO. 10.

### Original Contributions.

ARTICLE XXXV.

### ILEITIS. A CASE.

By S. P. BREED, M.D., of Princeton, Ill.

Master Charles E. Newell, aged 16 years, compositor, of temperate and studious habits, after complaining for several hours, sent for me on the 10th of July, ult., to see him.

Found him with some fever, which had been preceded by a chill. Pulse 80, moderately full and strong. His greatest complaint, however, was *pain* in the *bowels*, somewhat paroxysmal, though never quite easy. The bowels were very little distended, elastic, and rather harder to the feel than natural, with tenderness on pressure. The tongue was moist and slightly covered with a *dark*, slimy coating.

On inquiring whether he had eaten any cherries (as it was in the cherry season), he said that *he had.*

He also stated that there had been no motion from the bowels for 24 hours.

I concluded, from the symptoms and history of the case, that there had been imprudence in diet; and, as he had vomited freely, and his stomach, therefore, pretty well emptied, I ordered the following:—

℞. Hydrarg. Chlor. Mitis,---------------- grs. xx.
Ex. Colocy. Comp.,------------------- grs. xx.
Morphia Sulph., ----------------------- grs. j.

M. F't pulv., No. 5.

Sig. One every three hours.

Hop fomentations to the abdomen, and enemata of starch and laudanum.

℞. Tinct. Opii, ----------------------------- fl ʒss.

Mucilago Amyli,------------------------- fl ℥ij.

M. F't enema. Repeat every three hours, if retained, until he sleeps.

*July 11th.* Physic not operated. Vomited after each powder, except one or two. Injections generally retained. Abdomen much the same; uniform tenderness over the whole abdominal region. Distention moderate, but *general.* No tumor in ileo-cæcal region. Could trace the pain to no local point of starting. Pulse 90. Some delirium during sleep. Had rested pretty well. Symptoms much as yesterday, in all other respects. Ordered the following:—

℞. Hydrarg. Chlor. Mitis, --------------- grs. xx.
Ipecac, -------------------------------- grs. j.
Morphia Sulph.,---------------------- grs. ij.

M. F't pulv., No. 10.

Sig. One every three hours.

Enemata and fomentations continued.

*July 12th.* Physic not operated. Injections mostly returned unstained. Urine free, but not remarkably high colored. The vomiting persistent, decidedly bilious, and very dark. Pulse 98, soft, *rapid,* and undulating: artery large, and circulation languid. Copious perspiration, especially during sleep, which stood in bead-like drops on his forehead and face. Tenderness of the abdomen much the same. Not much distention, though quite uniform. No flatus or meteorism. On applying the ear to the abdomen no *sound was heard,* no gurgling; but, on the contrary, a dead silence prevailed throughout the whole abdominal region. It was now observed, also that, during the efforts at vomiting, the abdominal muscles did not coöperate with the thoracic and intercostal, but that they seemed utterly to refuse to act, which made his efforts to vomit peculiarly distressing. During the evening, the matter ejected by vomiting became of a grass-green color, and very acid, for which he took the following mixture:—

℞. Soda Bi-carb., ---------- ʒij.

Morphia Sulph., ---------- grs. iij.

Aqua Mentha, ---------- fl ℥iv.

M. F't sol. sig. One teaspoonful every two or three hours.

During the colliquative sweating, he took the following:—

℞. Quinia Sulph., ---------- grs. x.
Syr. Rhei. Arom., ---------- fl ℥j.

Sig. Teaspoonful every two hours.

At this stage of the case, I omitted the mercurial, under the impression that it tended greatly to the prostration of the patient, while I could see no benefits from its use, as desired. Until now, I had contented myself with opiates, fomentations, together with mercurials, hoping by these means to overcome the difficulty, by first *subduing* the *inflammation, which* I *thought* to be the prime cause of the constipation. As the mercurials had no effect (probably from not being absorbed), and, as there was great solicitude on the part of his friends, because there was no operation, I ordered the following:—

℞. Senna Alex., ---------- ℥j.

Coriander, ---------- ʒj.

Aqua Fervent, ---------- Oj.

M. F't, infusum. sig. Half an ounce every hour. The bowels were now bathed with the following:—

℞. Sweet Oil, ---------- ℥ij.

Sp'ts Ammonia, ---------- ʒj.

Croton Oil, ---------- ʒij.

M. F't liniment. sig. Apply to the bowels, and continued the fomentations, often renewed. Enemata with beef-tea added, continued at longer intervals.

*July 13th.* No operation yet. Vomiting continues. Sennatea thrown up. Countenance haggard. Expression anxious. Clothes quite wet with perspiration. Takes no nourishment, except the beef-tea in the enemata. Through this day he took ½ grain of morphia every two hours, and a dose of castor oil. His bowels were now bathed with hot oil of turpentine, and napkins wrung out of hot water were applied immediately, and this process repeated every half hour. Was called to the patient at 10 o'clock P.M. No operation; vomiting occasionally, matter ejected very dark; castor oil seen floating on the liquid. Has slept some. He now took 10 grains of calomel, with the hope that it would not be so easily rejected by the stomach, and might, therefore, be more likely to act as a physic than anything else that I could give. After waiting two or three hours, I determined to give an injection, sufficient to provoke an operation.

I thought of throwing this up high into the colon, through a bougie, but, as the injections had been generally retained, I felt sure that they had found their way up as high as I would be able to throw the fluid through the tube. Therefore, I omitted the tube, and injected some two quarts of soap, and tepid water, containing oil of turpentine and castor oil, well mixed together. This caused great pain, though he did not seem to realize where the pain was. When asked whether he did not desire to get up, he said, "Yes;" but when assisted to the sick chair, he made no efforts to procure an operation. No reflex action was called forth by the large enema, so as to induce straining; but, after sitting over the vessel some 10 or 15 minutes, part of the injection ran away, as clear as when thrown up.

I then order—

℞. Neutralizing Cordial, ------------------ ℥ij.
Morphia Sulph.,------------------------ grs. ij.

M. Sig. A teaspoonful every two hours. A large blister over the abdomen, and an enema of beef-tea and laudanum, occasionally, as before.

*July 14th.* Was summoned to his bedside at 10 o'clock A.M., where I met Dr. G. W. Crossley, in consultation.

There was less sweating than yesterday. Vomiting had continued occasionally. Countenance anxious—very restless. Blister not drawn yet. Passed urine several times. Pulse 100. Artery large, and felt soft and undulating under the finger, against which the pulse dashed with a kind of wave-like impression, and was gone. Prostration evidently increasing.

The consultation resulted in the exhibition of the following:

℞. Hydrarg. Chlor. Mitis, ................ grs. x.
Opium Pulvis, ........................... grs. iv.

M. F't pulv., No. 4. Sig. One every three hours, with directions to interpose between each powder ¼th grain of acetate of morphia, until he was tranquilized. The vesicant was reapplied. Seen again at 4 o'clock. Blister drawn well. Three or four ounces of feculent matter had passed the bowels, the first that had passed during his sickness. Vomiting continues even more distressing. Matter gulped up, without much straining, nearly black. Hickups occasionally. Pulse 110, soft, feeble, and undulating.

*July 14th.* Vomiting still continues. Several small fecal stools. Other symptoms much the same. Evidently failing.

All medicine *per orem* discontinued. Half a grain of the acet. of morphia applied to the epigastrium, after removing the cuticle. Beef-tea and laudanum enemata continued, in small quantities, at short intervals, with the view of thereby contributing something to the support of the failing strength; hoping (forlornly) that these fecal passages might be a harbinger of something more favorable, and an index of a gradual subsidence of the inflammation, and a relief of the obstruction.

*July 15th.* Vomiting continues, and has become stercoraceous. Urine passed several times. Pulse 120, soft, undulating, and quite compressible. Countenance hippocratic. Patient very restless, somewhat delirious, though he answers questions correctly. No meteorism; no borborygmi during the sickness; bowels slightly tympanitic. No effort at straining when he had a passage; on the contrary, whatever was injected ran away involuntarily in the bed, generally very little stained or colored. All hopes of the case were now given up, and nothing more really important was attempted in the way of cure; but it may be remarked that he took carbolic acid, properly diluted, with the view of correcting the fetor of the vomited matter, and it was also used freely in the utensils as a disinfectant, with much benefit.

The patient lingered on until 3 o'clock A.M., of the 16th, when he "passed that bourn whence no traveller returns."

This case had become one of great interest, from several considerations. The symptoms were so complicated that it was not possible to decide positively what the precise nature of the obstruction was. Moreover, only about two years before, Mrs. Newell had lost an older son, about the same age, with much the same symptoms.

This older son had the same obstinate constipation; all the symptoms of obstruction—stercoraceous vomiting, general tumefaction of the abdomen, tenderness, tympanitis, and the case, likewise terminated in death. He was treated, I understood, on the active cathartic non-mercurial plan, and finally was operated on by passing a bistoury into the abdomen, from which fecal matter issued through the wound. Before he died, however, (I was told) he passed fecal matter per *via naturales.* The history of the case had made a deep impression upon my mind; and when called to see Charles, I soon made up my mind that the chief difficulty proceeded, not so much from obstruction, as from inflammation in some portion of the alimentary canal; though I could discover no appearance of a tumor anywhere in the abdominal region, nor any accumulation of fecal or foreign matter, neither could I find, from the history, whether the pain had been at first local or not. Notwithstanding from a general survey of the whole case, the belief was strongly impressed upon my mind, from the absence of the *well-marked* symptoms of peritonitis, or from the superaddition of those that did not belong to peritonitis, that inflammation in the coats of the small intestines was the principal difficulty, and that this inflammation was to be met and subdued, not by *active cathartics*, but with *opiates*, *counter-irritants*, *revulsives*, and *mercurials.* Having thus made up my mind in regard to the pathology of the disease, and the proper mode to pursue, and having followed it with such skill and ability as I could command, and having thus signally failed, as well as the advocates of the heroic cathartic plan had done before me, it became a matter of the deepest interest to me, to look into this somewhat obscure matter a little further. Hence, it will not be surprising that I felt much gratified when I was requested to make a *post mortem* examination of the case. Two persons in one family thus dying of the same disease, and *that* a somewhat obscure one, was sufficient to overcome any objection that they might otherwise have felt, and even make them anxious to know precisely what the difficulty was.

Accordingly, at 11 o'clock A.M., a few hours after death, I met Drs. Crossley, Latimer, and Winter, for that purpose. Before proceeding to the examination, however, a few minutes were spent in giving a brief history of the case, its symptoms, treatment, and reasons for the examination.

It was stated that the case was one somewhat peculiar, as the symptoms did not make the differential diagnosis fully clear. It could not be considered an open, plain case of enteritis, as usually understood, with the inflammation commencing in the mucous membrane of the bowels, and thence extending to the other coats, from the fact that there had been no diarrhœa, at any time during the illness of the patient; but, on the contrary, there was constipation all the time—obstruction. It does sometimes happen that obstinate constipation and all the symptoms of obstruction occur after an attack of diarrhœa or cholera morbus.

A case occuring in the practice of the speaker, several years before, was referred to, wherein all the symptoms of impassable stricture, *strangulation*, occurred, running on to a fatal termination; after what had been regarded at first by an intelligent physician as Asiatic cholera. In this case, the inflammation undoubtedly commenced in the mucous coat of the bowels, and thence extended by contiguity of tissue to the other coats. This was not the course of the disease, however, in this case.

Again, there were, it is true, prominent signs of peritonitis; but, then, these signs were not well pronounced, and unmixed.

There did not appear in this patient the small, frequent, and corded pulse, which are the usual accompaniments of peritonitis; but, on the contrary, the pulse was slow, soft, and unresisting. There was not, moreover, that amount of distention and tympanitis usual in peritonitis. No suppression of urine. The tenderness was not especially marked here, nor was the position that of peritonitis, the patient lying, as often as any way, on his side, with no disposition to flex the legs upon the abdomen. There were, however, *general* pain, moderate tumefaction, uniform over the whole abdomen, not traceable to any focus, from which the inflammation had radiated to other parts and tissues. These symptoms might lead us to suppose the case one of *peritonitis*, if we could forget the still more prominent facts of obstinate constipation, persistent and most distressing retching, and vomiting of bilious, acrid, and stercoraceous matters, all of which declare this case to be something more than peritonitis.

Another unusual symptom, in this case, is the remarkable torpor, stillness, and *dead silence* in the abdominal cavity. No rumbling, no flatus, no borborygmi, no gurgling sound, indicative of action of the small intestines, which usually occurs in mechanical obstruction, especially in that portion of the tube above the impediment. But, in this case, there was a fearful ominous silence, which is a mystery to be explained, if possible, by the examination.

Reference was made to the different forms of disease causing obstruction, which should engage the attention of the practitioner, whenever called to a case with such symptoms as point to obstruction; and he will run them over in his mind, one after another, and compare each with the one he has on hand, to see whether or not they agree. The first one, perhaps, that will be considered will be hernia. Having laid this over as not the one he has to treat, he next probably thinks of intus susception, intestinal concretions, volvulus, stricture from inflammatory adhesions, or thickening of the parietes of the bowels, and a consequent narrowing down of the caliber of the tube. Organized bands thrown across the passage. Such bands probably originate in an inflammatory adhesion of the surfaces of the mucous membrane, in consequence of the effusion of coagulable lymph, and a subsequent separation of these surfaces before the lymph becomes consolidated, so that it is drawn out into apparently interlacing cords. Twisting of the bowels, so that a loop of the small intestine gets turned around upon itself, is another cause of fatal obstruction. This occurs sometimes from injuries, colic, etc. Still another cause of obstruction is strangulation, by the passage of a loop of the bowel through some abdominal opening, as, for example, through a rent in the mesocolon. Organized tumors are also sometimes found exterior to to the bowels, which, by pressure upon them, so diminish the caliber of the passage as to produce the obstruction. These are some of the principal causes which may produce obstruction; and although it may not be apparent in all cases, during life, as to which one, if any, of these causes occur in any particular case, yet, it is believed, that in most cases a careful investigation of all the symptoms, together with the whole history, will enable the physician to make out a pretty correct opinion. But, in the case before us, it was remarked, that a decided opinion could not be formed as to which one, if any, of the above classes it belonged. Hence the necessity for the examination.

On opening the abdomen, which was moderately distended, the peritoneum was found to be thickened, softened, and its bloodvessels enlarged, of a very dark color, but no effusion into its cavity. The small intestines (especially the jejunum) were nearly empty, *though not collapsed*, but generally discolored, from the effects of the previous inflammation, with extensive adhesions agglutinating them together in various places, and in abnormal positions.

The muscular coat of the small intestines, and more especially that in the region of the illeo-cæcal valve, was very dark, thickened, softened, and nearly in a state of sphacelus.

The mucous coat here was also thickened, softened, of an ash color, and thrown into rugous bands encircling the intestine, between which there seemed to be a solution of continuity, through which the sub-mucous and muscular tissues could plainly be seen, of a deep purple color. See specimen.

Stomach not opened, but its external appearance seemed healthy. And, as the intensity of the inflammation in the small, intestines increased as we passed down towards the lower portion, it was not thought necessary to examine the stomach further. Liver healthy. Gall-bladder moderately distended with healthy bile. The other organs examined were healthy.

There was no mechanical obstruction. No apparent impediment to the passage of the fecal matter, which was, moreover, soft and nearly fluid, and, therefore, altogether favorable to such passage. The bowels did not appear to be overdistended, at any point, with fecal, crude, or undigested matters. No impacted feces; no foreign substances lodged in any portion of canal, which could have produced the symptoms of obstruction. What then was the cause of the above symptoms? What was the pathology of the case? And what important practical lesson may be drawn from the facts before us?

It will be seen at once that this is a very interesting and instructive case. The difficulty was not found to be mechanical obstruction, from the retention in the bowels of any improper ingesta. Was it peritonitis? The symptoms did not pronounce it as such. Though there was peritonitis, there was, at the same time, *something more.* Was it enteritis? No, not simply enteritis, as usually defined in the books. It will be seen, therefore, that the symptoms and anatomical changes mark the case as *sui generis.*

It was not a case of obstruction from the effusion of lymph among the tissues of the intestinal walls; not intussusception volvulus; nor yet intestinal concretions of any kind. It was not a case of exantheme interne of the French, neither enteritis, nor yet typhlitis stercoralis, nor perityphlitis; as in these cases there would have been *at first pain* and *tumefaction* in the right hypochondrium, with a distinct hardness in this region, with, probably, symptoms of pressure upon the nerves, as they pass down to the right lower extremity, such as numbness, etc. These diseases are all excluded from the list, both by the symptoms and morbid anatomy in the case.

What, then, was the disease? This is the question. It was

*inflammation* of *all* the coats of the small intestines, extending nearly through the whole length of the jejunum, ileum, and cæcum.

Therefore, it may be declared that the obstinate constipation was the cause of the gastric irritability, by the operation of a well-known law of remote sympathy subsisting between distant portions of the alimentary tube. But what caused the constipation and symptoms of obstruction, it may be asked? Mucous inflammation? No. Peritoneal? No. We must look further than to the ordinary results of inflammation in these structures to find the explanation. What more have we left? The sub-mucous and the *muscular coats.* But these structures do not appear as distinct diseases in any of our learned works, on the diseases of the abdominal *viscera.*

It seems a little strange, when the importance of the function of the *muscular coat* is considered, that no distinct disease of this structure is described in our medical works. Can any one explain the reason for this hiatus? In the light of the pathology and symptomatology of this case, then, we will venture to *declare*, with much confidence, and, indeed, we would give *great emphasis* to the *fact*, that *inflammation* of the *muscular coat* of the small intestines is fully capable of producing *all* the *symptoms* of *obstruction*—strangulation. The implication of *this coat* is the cause of the superaddition of those symptoms which could not be accounted for otherwise.

The obstinate constipation, bilious, acrid, and even stercoraceous vomiting, it is believed, were, in this case, caused by the inflammation of the muscular coat of the bowels.

How did it do this? By arresting the function of the *muscular fibers* of the intestinal walls. *Inflammation* in these *circular fibers*, by suspending the normal molecular movements necessarily involved in their functional activity, and thus inevitably interfering with that sympathetic, consecutive, coördinate contraction of these fibers, which are the efficient, and, indeed, the only agents engaged in the propulsion of the alimentary mass through the intestinal tube, *will as surely* arrest the *vermicular motion* and *peristaltic action* of the *bowels* as any *concrete*

*mechanical obstruction* whatever, and will, therefore, give rise to all the symptoms of obstinate *constipation*, *physical obstruction*, and *impassable stricture.* This is the only hypothesis that fully explains all the foregoing group of symptoms. The dead silence; the significant, profound torpor of the small intestines can be accounted for in no other way. But it may be asked whether inflammation in these fibers is competent to produce this result? Let us see. When inflammation is set up, either in the peritoneal or mucous coat of the bowels, without extending into the muscular coat, we generally have diarrhœa. This diarrhœa is induced by an excitement of the excito-motory system of nerves, through reflex action. Thus, the muscular fiber is called into activity by the stimulus of a *contiguous inflammation*, and not from inflammation in its own tissue. It is a conservative remedial, and purely a physiological phenomenon, looking to the removal of the cause of the difficulty. But as soon as the *inflammation* extends into the substance of the *muscular tissue itself*, there will be, *not diarrhœa*, but *constipation instead.* I have already given one case, and I might give many more. It is, however, quite unnecessary, as all that is needed is simply to direct attention to the fact, and many instances illustrating this point will immediately spring to the mind. A muscle *inflamed* is not only indisposed, indeed, incompetent to act; but a forced action will be sure to increase the pain and aggravate the inflammation. If these statements are true, which, I doubt not, will be admitted, then this case becomes a most significant and important one to the medical philosopher, as it furnishes one more of the few cases recorded in the annals of medicine and pathology, to demonstrate the fact, that *inflammation* of the *muscular coat* of the bowels (which has been sadly overlooked) is fully competent to produce all the symptoms of strangulation.

The above is a good case in point to illustrate the practical bearings of this question. Had the case been regarded as one of mechanical obstruction, from the impaction in the canal of some foreign matter as the prime cause of the difficulty, and more vigorous measures employed, active cathartics brought into requisition, it is *now* easy to see that such a course (which, it is feared, is too often pursued in similar cases) must have greatly aggravated the symptoms and diminished the chances of recovery. Let the practitioner carefully examine his cases of obstruction, and study well the differential diagnosis; and, if he have good reason to believe that the inflammation has its seat in the *muscular coat* of the bowels, either from the extension of peritonitis or enteritis, let him be careful how he tampers with active cathartics. On the contrary, let him put the bowels at rest; put them in splints, as it were, on the same principle that he would an injured limb; give them repose and time to recover; while, by the timely and judicious use of opiates, revulsions, *mercurial alteratives*, and other appropriate means, let him seek *first* to subdue the *inflammation* in these delicate tissues, so that they may be released from *restraint*, and *they* will *take care* of the *constipation*.

We have here, then, a notable case, where a considerable portion of the alimentary tube is so *paralyzed*, from the effects of *inflammation*, *involving all the coats* of the *bowels*, that all normal and physiological function is necessarily suspended between it, and the general system, responding to no medicines, awakened by no ordinary sympathies, controlled by a fatal synergy, bidding defiance to all available modes of treatment, and throwing a fearful spell, like some magic wand, over all the other functions and organs of the body, and drawing them with an irresistable power into its fatal toils. As we stand by and witness the progressive steps of this disease, as it advances unerringly to the very citadel of life, it requires no great stretch of fancy to imagine the system struggling in the iron grasp of some unseen antagonist, writhing in fearful agony, and straining every nerve to break the spell of the spoiler, and escape from his fatal meshes; but, like the fabled Prometheus, chained fast to the rock, while the vulture preyed continually on his liver, all efforts were unavailing; while the hidden foe "like a staunch murderer," steady to his purpose, redoubling his exertions, casts still further his mortal coils around his helpless victim, tightens more firmly his determined grasp, until one after another of the struggling, quivering members are bound firmly, and fast, *pinioned*, "as with hooks of steel," beyond the possibility of escape; so that, further resistance being impossible, the only alternative left is a tame submission to the irresistible power of a hopeless destiny.

We can *now* understand *why* there was no *movement* of the *bowels*, no, sign of *action* or *life*. Why that *dread* silence and awful stillness prevailed in the abdominal cavity, when all other organs were strained up to their utmost tension.

A fearful *inflammation*, *sudden*, *insidious*, and *destructive*, had seized, as with the hand of some etherial Titan, upon this portion of the body, arresting at once all the normal actions therein—muscular contractility destroyed, absorption through the thickened membrane stayed, molecular movements obliterated, and the nutritive function held in abeyance, until all the vital forces are brought to a dead lock, or converted into so many fierce enemies to human life and mortal flesh, to rankle and riot in their delectable work of destruction, disease, and death.

Such is the fate of all flesh.

